# Mining SNP loci and candidate genes for sheath blight resistance in *Indica* rice using genome-wide association studies

**DOI:** 10.3389/fpls.2025.1718389

**Published:** 2025-12-18

**Authors:** Xin Gu, Junjie Ding, Hui Xu, Wei Liu, Hui Wu, Qiang Tang, Shuai Yang, Ling Wang, Suhua Zhang, Long Chen, Xue Meng, Ye Tao, Jing Hao, Zhao Chen, Shuhua Jiang, Ruoting Yu, Liying Xu, Muhammad Ahmad Hassan

**Affiliations:** 1 College of Landscape Architecture and Horticulture, Wuhu Vocational University, Wuhu, China; 2 Jiamusi Branch of Heilongjiang Academy of Agricultural Sciences/ Observation and Experiment Station of Crop Pests of Jiamusi, Ministry of Agriculture and Rural Affairs, Jiamusi, Heilongjiang, China; 3 Shenyang Polytechnic College, Shenyang, China; 4 College of Resource and Environment, Anhui Agricultural University, Hefei, China

**Keywords:** *oryza sativa* subsp. *indica*, sheath blight, genome-wide association study, resistanthaplotype, disease resistance genes

## Abstract

Rice is a vital component of the global food supply chain. Sheath blight (ShB) poses a severe threat to *indica* rice production, resulting in substantial yield and quality deteriorations. Although chemical control strategies are widely employed, their prolonged use raises concerns about the development of pathogenic resistance. Genome-wide association studies (GWAS) serve as a critical tool for identifying genetic loci associated with ShB resistance. However, current research efforts are constrained by limitations in sample size, environmental influences, and analytical methodologies. Notably, studies on ShB resistance have focused mainly on *japonica* rice, with *indica* rice receiving limited attention in GWAS-based investigations. Prioritizing GWAS research targeting ShB resistance in *indica* rice is essential for enriching disease resistance gene resources and facilitating molecular breeding programs. Eighty-four *indica* accessions were subjected to *in vitro* phenotyping for ShB resistance, followed by whole-genome resequencing using a magnetic bead-based DNA extraction protocol. Population structure analysis revealed high genetic diversity (K = 5) with normally distributed resistance phenotypes. From 2.93 million raw SNPs, 904,708 high-quality variants were retained for multi-model GWAS (GLM/MLM/EMMAX/GEMMA), identifying 22 consensus loci. Haplotype blocks delineated three critical regions harboring 15 candidate genes, including ABC transporters and calmodulin-like proteins with putative roles in pathogen defense, among others. Functional annotation revealed that ABCG transporters, calmodulin-like proteins (CMLs), non-specific lipid transfer proteins (nsLTPs), and receptor-like serine/threonine kinases orchestrate defense responses through calcium-mediated signaling, antimicrobial metabolite translocation, and maintenance of membrane integrity. qPCR analysis revealed that the three GWAS-associated candidate genes were significantly upregulated after *R. solani* inoculation in the resistant line X-11, suggesting that variations in their non-coding regions contribute to disease resistance by enhancing both basal and pathogen-induced expression. These loci constitute a genetic toolkit for deciphering *Rhizoctonia solani* Kühn (*R. solani*) resistance mechanisms and facilitate precision breeding in *indica* rice.

## 1 Introduction

Rice (*Oryza sativa* L.) is a crucial component in ensuring global food security, as it fulfills the dietary needs of half of the world's population (Hassan et al., 2023; Zhu et al., 2024). Rice productivity is endangered by several biotic (pathogenic diseases) and abiotic factors (temperature extremes, heavy metals, salinity, drought, etc.) (Turaidar et al., 2018; Khoshkdaman et al., 2021). Among biotic factors, diseases (i.e., blast, blight, etc.) are significant constraints for yield and quality reduction in rice (Turaidar et al., 2018). Rice sheath blight (ShB) is a prevalent and frequently occurring disease in rice, causing substantial yield reductions (20~50%), particularly in *indica* rice (*Oryza sativa* ssp. *indica*) (Richa et al., 2016; Srivastava et al., 2024). The *Rhizoctonia solani* Kühn (*R. solani*), a soil-borne fungal pathogen, is the causative agent of rice ShB (Ganesh and Roka, 2024). It was first identified in Japan and subsequently detected in other rice-producing regions, including China, Bangladesh, Vietnam, Thailand, Pakistan, and others (Srivastava et al., 2024). Prolonged high humidity (>80%) and temperatures of 28~35°C are favorable conditions that accelerate epidemic development of rice ShB (Uppala and Zhou, 2018). Long, uneven, greyish green spots with brown margins on leaves and developing panicles are the symptoms of rice ShB, which is also known as snakeskin disease due to its symptoms resemblance with snakeskin (Molla et al., 2020; Hunjan et al., 2022) Fungicide application remains a primary control strategy, its overuse has triggered the widespread evolution of resistant *R. solani* strains, as it remains dormant under the soil for up to 2 years and become active on favorable conditions, such conditions jeopardizing the sustainable rice cultivation systems (Hunjan et al., 2022; Ganesh and Roka, 2024; Zhai et al., 2025).

Genome-wide association studies (GWAS), empowered by high-density genotyping and next-generation sequencing, have revolutionized the dissection of polygenic trait architectures in plant systems (Chen et al., 2014; Wang et al., 2020). GWAS has proven instrumental in decoding the genetic architecture of ShB resistance, with recent advances enabling systematic mining of resistance-associated alleles (Zhang et al., 2019). The widespread infestation of ShB in *indica* rice-growing regions becomes more severe due to humid climatic conditions that are conducive to *R. solani* proliferation (Yu et al., 2025). This epidemiological pressure has driven rigorous efforts to identify ShB-resistant genetic loci and utilize them for developing elite cultivars, a strategic priority in contemporary disease-resistance breeding plans (Hunjan et al., 2022). Advances in high-throughput sequencing and population genetics algorithms have substantially enhanced the power of GWAS in decoding the genetic complexity of rice ShB resistance (Lu et al., 2025). Wang et al (Wang et al., 2021). have conducted an investigative study and performed the whole-genome resequencing of 259 rice accessions, integrating SNP-GWAS, haplotype-based GWAS (Hap-GWAS), and weighted gene co-expression network analysis (WGCNA) to identify 653 core candidate genes associated with ShB resistance. Multi-environment trials conducted across diverse locations and seasons strengthened the ecological validity; however, the inherent environmental heterogeneity of field conditions may introduce confounding factors, potentially obscuring the causal relationships between genotype and phenotype. Building on this methodological consistency, subsequent research analyzed 563 rice accessions from 47 countries, representing *indica*, *japonica*, and *aus-type* subpopulations (Zhang et al., 2019). Utilizing ~3 million SNPs, they employed both single-locus GWAS (EMMAX) and multi-locus approaches (FarmCPU) to dissect ShB resistance traits, i.e., culm length (CL), lesion height (LH), and relative lesion height (RLH). Cross-validation between methods enhanced the reliability of identified resistance-associated loci (Chen et al., 2019). Complementing these multi-model approaches, a subsequent investigation conducted genotyping of 228 *indica* accessions using a high-density rice SNP array (700K markers), identifying multiple significant loci associated with sheath blight resistance on chromosomes 1, 4, and 11 through GWAS (Oreiro et al., 2020). The study further integrated transcriptomic profiling with GWAS signals to elucidate resistance mechanisms across both positional genomics and gene regulatory dimensions. This dual-system validation strengthens mechanistic plausibility beyond statistical associations. Echoing this multi-system strategy, Li et al (Li et al., 2022). implemented a multi-environment trial framework that combined field evaluations in Arkansas (USA) and Nanning (China) with greenhouse micro-chamber assays to dissect ShB resistance alongside agronomic traits, including plant height, heading date, tiller number, and panicle count. GWAS identified 22 ShB-associated SNPs across chromosomes (excluding Chr10 and Chr12), with 13 intragenic SNPs encoding proteins such as protease inhibitors, ATPases, and cyclins, several of which exhibit pathogen-responsive functions and have been validated in prior studies. Notably, seven resistance loci co-localized with previously reported ShB resistance genes or quantitative trait loci (QTLs), reinforcing their biological relevance. However, environmental heterogeneity (divergent climates and soils) and distinct pathogen isolates across varying test locations complicate the precise resolution of genotype-by-environment (G × E) interactions. Building on this genetic dissection, Chen et al (Chen et al., 2019). utilized the Rice Diversity Panel 1 (RDP1, comprising 299 accessions and 44K SNPs) to elucidate ShB resistance through controlled greenhouse inoculation and standardized phenotyping. GWAS revealed 11 significant loci, with two robust QTLs (i.e., *qSB-3* and novel *qSB-6*) on chromosomes 3 and 6, respectively. Haplotype analysis demonstrated additive effects: accessions carrying dual superior haplotypes (AGC) exhibited a 1.21-unit reduction in lesion score. Particularly, the aromatic (ARO) and aus subgroups displayed higher resistance than the tropical *japonica* (TRJ) and *indica* (IND) subgroups. However, the limited sample size of the ARO subgroup (n = 11) may have constrained statistical validation.

Whereas GWAS generally offer stronger statistical authentication in large populations, identifying disease-resistant loci with limited samples is feasible via optimized experimental designs and methodological improvements (Wang et al., 2019; Uffelmann et al., 2021). A genomic analysis conducted GWAS on 150 accessions from the Ting core collection genotyped with 5.1 million SNPs (Fu et al., 2022). While phenotyping across two seasons (2016-2017) aimed to validate the robustness of the results, the limited cohort size constrained statistical power and generalizability. Notably, no SNPs showed replicable associations across years, underscoring the environment-dependent nature of resistance mechanisms. Similarly, another research investigation employed a multi-locus GWAS framework (FarmCPU/BLINK) in 96 sweet potato genotypes to identify Fusarium root rot resistance loci, implementing stringent false discovery rate control (Kim et al., 2023). Cross-model validation, combined with Bonferroni-adjusted thresholds, reduced spurious associations compared to single-model approaches, demonstrating methodological triangulation as an effective strategy to enhance GWAS reliability in small cohorts (n < 100). This combinatorial strategy has parallel applications in research, where GWAS on 144 maize accessions (using the Illumina MaizeSNP50 array) identified 18 head smut resistance genes (Wang et al., 2012). To mitigate cohort size constraints, the study implemented threefold optimization: 1) strategic selection of genetically diverse germplasm; 2) rigorous quality control pipelines; 3) multi-model validation using general and mixed linear models (GLM/MLM). Such methodological layering effectively compensates for population-scale limitations while maintaining robustness in the discovery process.

Furthermore, all existing studies have demonstrated that cohort size, experimental conditions, and analytical methodologies significantly influence GWAS outcomes. While large populations enhance statistical authenticity, practical constraints—such as the minimal availability of germplasm with specific genetic backgrounds—often restrict sample sizes. Small-cohort GWAS can achieve biological relevance through methodological safeguards: multi-model association analyses, stringent significance thresholds, population structure correction, and kinship matrix integration. Notably, current ShB resistance research disproportionately focuses on *japonica* rice, leaving *indica* systems underexplored. Building upon these insights, our study utilizes a localized *indica* core collection to dissect the architecture of ShB resistance. Employing Tassel-based GWAS with minor allele frequency (MAF) filtered SNPs (>0.05), we integrate GLM/MLM to identify consensus loci. Subsequent linkage disequilibrium (LD) block delineation and candidate gene mining within critical regions refined the mechanistic hypotheses.

## 2 Materials and methods

### 2.1 Plant material and phenotypic evaluation

The plant material of 84 *indica* rice accessions was used in this research experiment. Detailed information about the breeding background and geographical distribution of these 84 accessions has been presented in **Supplementary Table S1**. Rice ShB resistance phenotyping of 84 accessions was performed using an optimized *in vitro* inoculation assay adapted from Li et al (Li et al., 2022). Additionally, 20 accessions were randomly selected, and the results were validated through field-based toothpick insertion inoculation. Locally isolated *R. solani* strains were cultivated on potato dextrose agar (PDA) medium, with 5-mm mycelial plugs serving as inoculum. Healthy leaves from four-leaf-stage seedlings were surface-sterilized and excised into 6 cm segments. Four segments per plate were arranged on triple-layer sterile filter paper in 9 cm Petri dishes, centrally inoculated with mycelial plugs, and maintained at 25°C under a 12 h photoperiod. To sustain humidity, a pH-adjusted 40 mg/L 6-BA solution was applied. Disease severity was recorded at 24 h post-inoculation using a 6-tier scale, as follows:

- 0 (Immune, I): No lesions
- 1 (Resistant, R): <1/8 leaf area affected
- 2 (Moderately resistant, MR): 1/8–1/4
- 3 (Moderately susceptible, MS): 1/4–1/2
- 4 (Susceptible, S): 1/2–3/4
- 5 (Highly susceptible, HS): >3/4

Three biological replicates were conducted for each accession.

### 2.2 DNA extraction and resequencing

Genomic DNA was isolated from 84 accessions using a magnetic bead-based extraction method. DNA concentration and purity were verified using Nano-Drop 2000 (Thermo Fisher Scientific). Sequencing libraries were prepared through fragmentation, size selection, and adapter ligation and then sequenced on an Illumina NovaSeq 6000. Raw reads were filtered (Phred score < 20) and aligned to the reference genome (MSU v7.0) using Bowtie2 v2.3.5.1 (Langmead and Salzberg, 2012), with subsequent sorting performed via SAMtools v1.3.1 (Danecek et al., 2021). Redundant reads were filtered using Mark-Duplicates from Picard (Belay et al., 2024), followed by variant calling via GATK4 Haplotype-Caller (McKenna et al., 2010). Joint genotyping generated population-level genomic variant call formats (gVCFs), filtered through stringent quality thresholds (QUAL ≥30, QD ≥2.0, MQ ≥40, FS ≤60.0, MQRankSum ≥-12.5, ReadPosRankSum ≥ 8.0). Final SNP sets underwent MAF (≥0.05) and missing rate (≤20%) filtering using VCF-tools v0.1.6 (Danecek et al., 2011), with genetic diversity assessed via PLINK toolkit (Purcell et al., 2007).

### 2.3 Population analysis and structure analysis

Following variant filtering, population genetic analyses were conducted using TASSEL5.0 (Bradbury et al., 2007). Principal component analysis (PCA) and kinship estimation were employed to identify genetic relationships among the 84 *indica* accessions. A neighbor-joining (NJ) phylogenetic tree was constructed and visualized in MEGA11 (Tamura et al., 2021). Population structure analysis using ADMIXTURE (Alexander et al., 2009) identified K = 5 as the optimal clustering parameter, as determined by minimizing the cross-validation error, with corresponding ancestry proportions graphically represented.

### 2.4 GWAS analysis and linkage disequilibrium analysis

To enhance statistical robustness in GWAS with limited sample size, we implemented a multi-model framework integrating TASSEL's GLM, MLM, EMMAX (Kang et al., 2010), and GEMMA's (Zhou and Stephens, 2012) Bayesian sparse linear mixed models. The LD decay analysis via PopLD decay (Zhang et al., 2019) informed recombination boundaries. Significant phenotype-associated SNPs were cross-validated across models, with LD blocks delineated through LD-Block-Show visualization. Candidate genes within these blocks were prioritized through functional annotation against established databases of plant-pathogen interactions. The functions of the candidate genes were annotated using the NCBI (https://www.ncbi.nlm.nih.gov/), UniProt (https://www.uniprot.org/), and Plant-Pathogen Interactions (http://www.phi-base.org/) databases.

### 2.5 Quantitative real-time PCR analysis

Three ShB resistance candidate genes (*BGIOSGA000756*, *BGIOSGA036403*, and *BGIOSGA037213*) previously identified through GWAS were selected for expression analysis. The resistant line X-11 and susceptible line X-71 were inoculated with *R. solani*, and leaf samples were collected at 0 and 24 hours post-inoculation (hpi). Total RNA was extracted from leaves using the MiniBEST Plant RNA Extraction Kit (TaKaRa). RNA quality and quantity were assessed, and qualified RNA was reverse-transcribed into cDNA using the PrimeScript™ 1st Strand cDNA Synthesis Kit (TaKaRa). Gene-specific primers for each candidate gene are listed in **Table 1**.

**Table 1:** Primer sequences used for qPCR.

	Gene	Forward primer	Reverse primer
1	*BGIOSGA000756*	AGATCTTCCGCCACTTCGAC	TCGTAGGCGCTTATCATGG
2	*BGIOSGA036403*	GGTCAATGGAATCCCTGAG	ACAATGCTCCTGTGTGGAC
3	*BGIOSGA037213*	ATTAGTGTGAGTGCTGTGGTG	TGGCTGCTTGTGGATTTAG

Quantitative real-time PCR (qPCR) was performed using the SupRealQ Ultra Hunter SYBR qPCR Master Mix (U+) (Vazyme Biotech) on an MA-6000 Real-Time PCR System (Suzhou Yarui Biotechnology). Each 20 μL reaction contained 10 μL of 2× Master Mix, 1.0 μL of cDNA template, and 0.4 μL each of forward and reverse primers (10 μM). The thermal cycling conditions were as follows: initial denaturation at 95°C for 5 minutes, followed by 40 cycles of 95°C for 15 seconds and 60°C for 30 seconds. The rice Actin gene (*BGIOSGA033259*) was used as an internal reference for normalizing relative gene expression, with the following primer sequences: forward, 5‘-GCCATTCTCCGTCTTGATCTTGC-3'; reverse, 5‘-AGCGACAACCTTGATCTTCATGCT-3' (Baiya et al., 2014). Relative gene expression levels were calculated using the 2–ΔΔCt method. The statistical significance of expression differences was determined using an independent-samples t-test, with *p* < 0.05 considered significant and *p* < 0.01 considered highly significant.

## 3 Results

### 3.1 Phenotyping

Dual *in vitro* ShB resistance assays yielded statistically concordant phenotypic measurements, as determined by density plot analysis (**Supplementary Figure 1**). Consequently, the lesion length ratio was selected for genome-wide association studies. The *indica* panel (n = 84) exhibited a resistance spectrum stratification, with 8 resistant (R), 17 moderately resistant (MR), 51 moderately susceptible (MS), and 8 susceptible (S) accessions. The normal distribution of resistance phenotypes (**Supplementary Table S2**), characterized by the predominance of moderate resistance and lack of extreme phenotypes, reflects the natural population architecture and validates its suitability for GWAS. Importantly, all genotypes displayed measurable lesions, including nominally resistant lines, confirming successful pathogen invasion. The results showed that the coincidence rate between toothpick insertion inoculation and *in vitro* leaf inoculation at the tillering stage was high, reaching 85%, indicating that seedling-stage *in vitro* leaf inoculation can accurately identify phenotypes (**Supplementary Table S3**, **Supplementary Figure S1**, **Supplementary Figure S2**).

### 3.2 Genotyping

**Figure 1:**
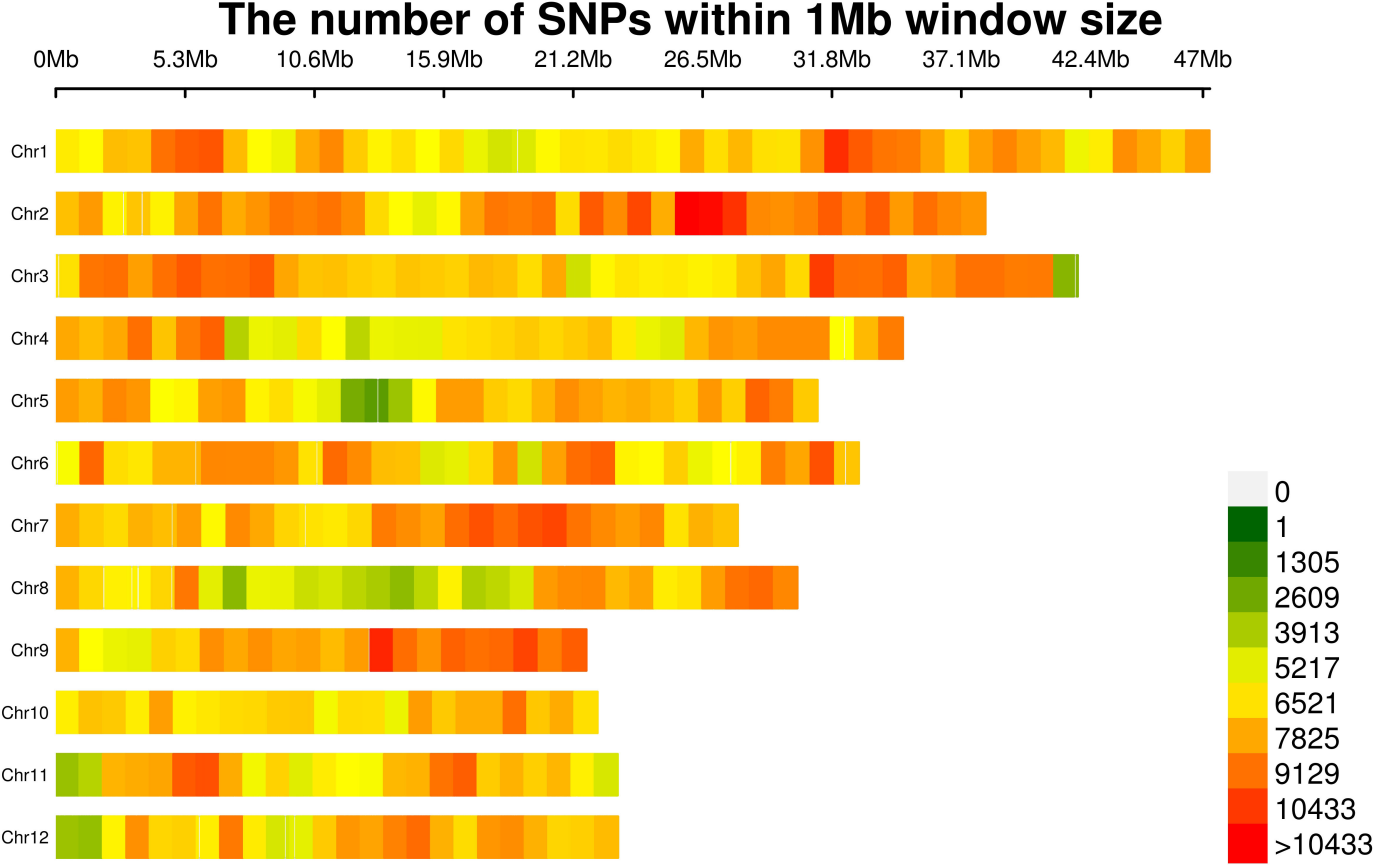
Distribution of single-nucleotide polymorphism (SNP) density across *indica* rice chromosomes. The vertical axis represents the 12 chromosomes of *indica* rice, while the horizontal axis denotes chromosome length. The color scale indicates the number of single-nucleotide polymorphisms (SNPs) within 1.0 megabase (Mb) windows.

Variant calling and stringent filtering through the Genome Analysis Tool Kit (GATK) yielded 2,933,319 genome-wide SNPs (**Table 1**), distributed across all 12 chromosomes and scaffolds (**Figure 1**). Chromosomal SNP densities ranged from 6.43/kb (Chr8) to 8.57/kb (Chr2), averaging 7.48 SNPs/kb (**Supplementary Table S4**). Post quality control (QC) metrics revealed minimal missing data (<15% per sample) and pronounced allelic asymmetry: 65.28% of SNPs exhibited MAF <0.05, with heterozygosity levels below 0.15 in 98.82% loci; patterns are consistent with rice's autogamous reproduction. Final GWAS-ready SNPs (904,708) were obtained through MAF (≥0.05) and missing rate (≤20%) filtration. As shown in the supplementary results, the average sequencing depth of the 85 indica rice accessions ranges from 4.53X to 13.54X (mean = 9.02X); the 1X sequencing depth coverage rate varies between 0.68 and 0.87 (mean = 0.80); and the 5X sequencing depth coverage rate is from 0.44 to 0.77 (mean = 0.67). Although minor variations exist in the sequencing coverage metrics among different accessions, the resequencing data overall exhibit good genome coverage completeness. This reliable dataset can provide solid support for subsequent studies, such as genetic diversity analysis and functional gene mining (**Supplementary Table S5**).

### 3.3 Population structure analysis

**Figure 2:**
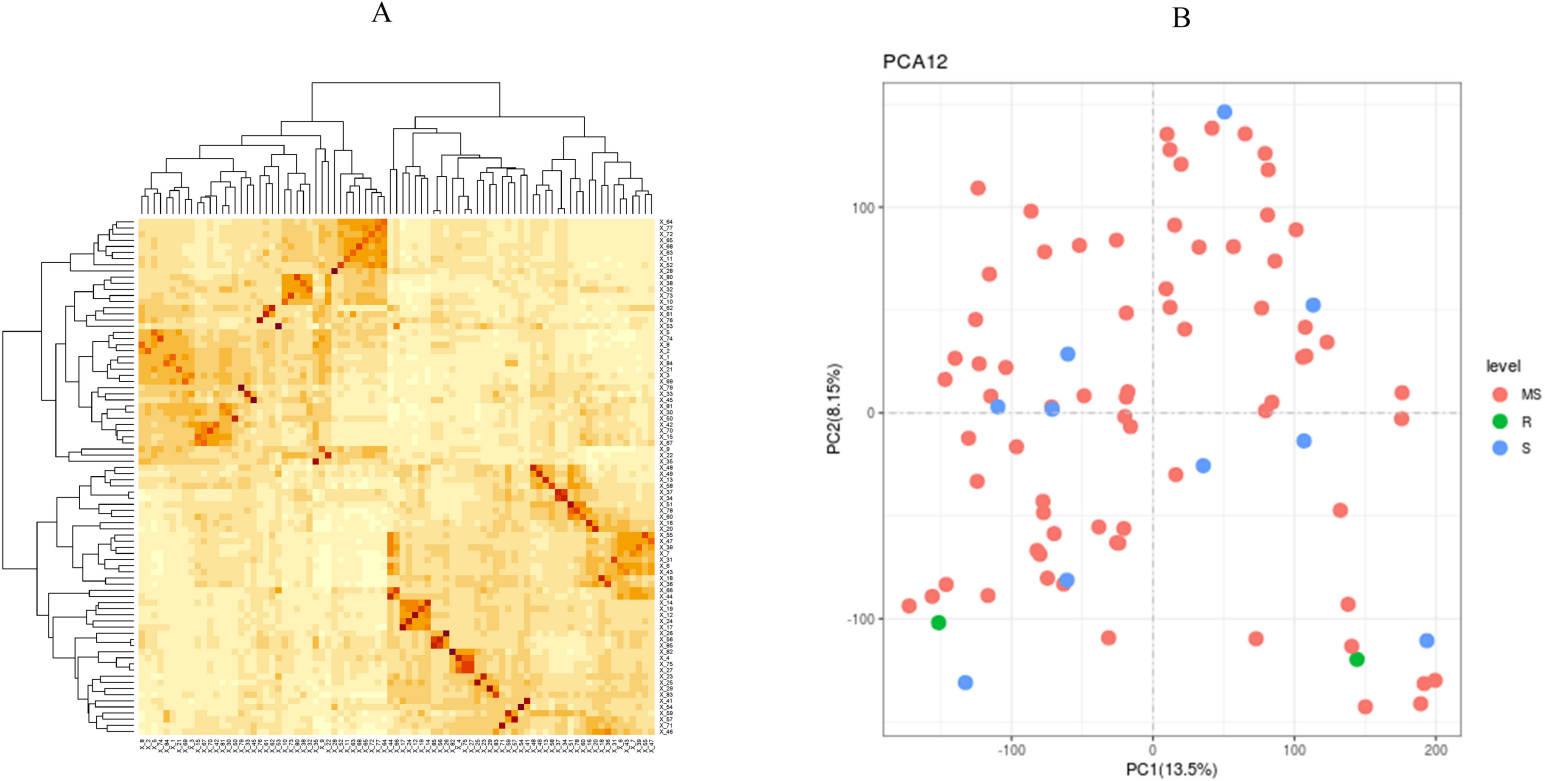
Genetic structure and phylogeny of the population. **(A)** Principal component analysis (PCA). **(B)** Heatmap of pairwise relative kinship estimates.

**Figure 3:**
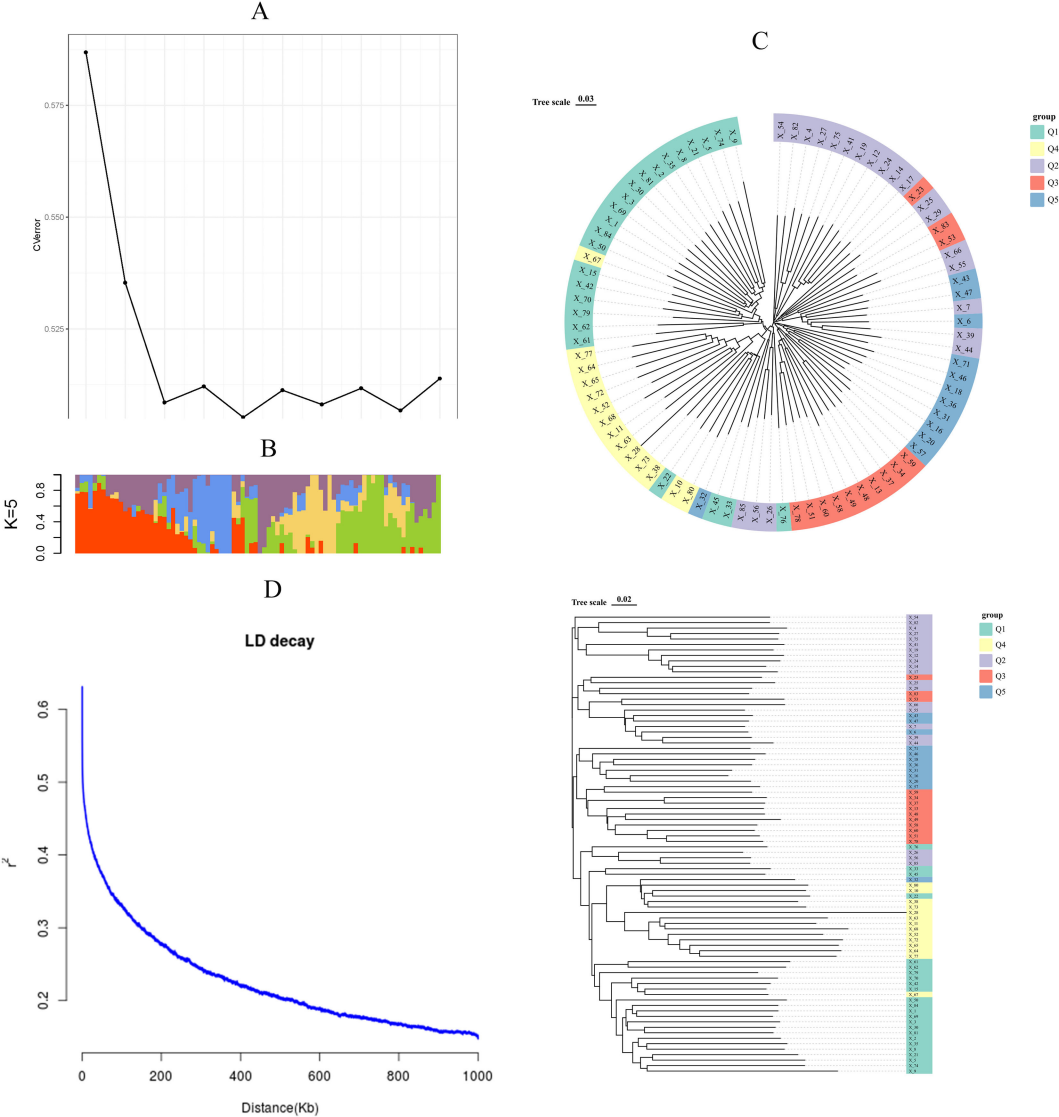
Population Genetic Architecture and LD Decay Analysis. **(A)** Cross-validation error plot for determining optimal ancestral clusters (K = 5). **(B)** ADMIXTURE bar plot showing genetic structure across 84 accessions (K = 5), with colors representing distinct ancestral components. **(C)** Neighbor-joining phylogenetic tree color-coded to ADMIXTURE clusters, demonstrating weak population substructure. **(D)** genome-wide linkage disequilibrium (LD) decay profile showed that the LD of the population decayed to half its initial value at an approximate distance of 70 kb, with a corresponding r² of approximately 0.35.

Among 84 *indica* accessions, there was a little substructure found in the population genetic analyses of 904,708 SNPs Principal component analysis (PCA) demonstrated no disease resistance-based clustering (**Figure 2A**). In contrast, kinship heatmap analysis identified localized regions of moderate relatedness without distinct population stratification (**Figure 2B**). ADMIXTURE analysis determined K = 5 as the optimal cluster number through cross-validation error minimization (**Figure 3A**). However, ancestral components showed extensive admixture across accessions (**Figure 3B**). Phylogenetic re-construction via neighbor-joining method yielded short branches with overlapping clusters across K = 5 subgroups (**Figure 3C**), validating PCA/kinship findings of high genetic diversity and low differentiation. The LD decay analysis revealed moderate recombination rates, with the LD decay distance at ~70 kb (**Figure 3D**).

### 3.4 GWAS of genes resistant to ShB

**Figure 4:**
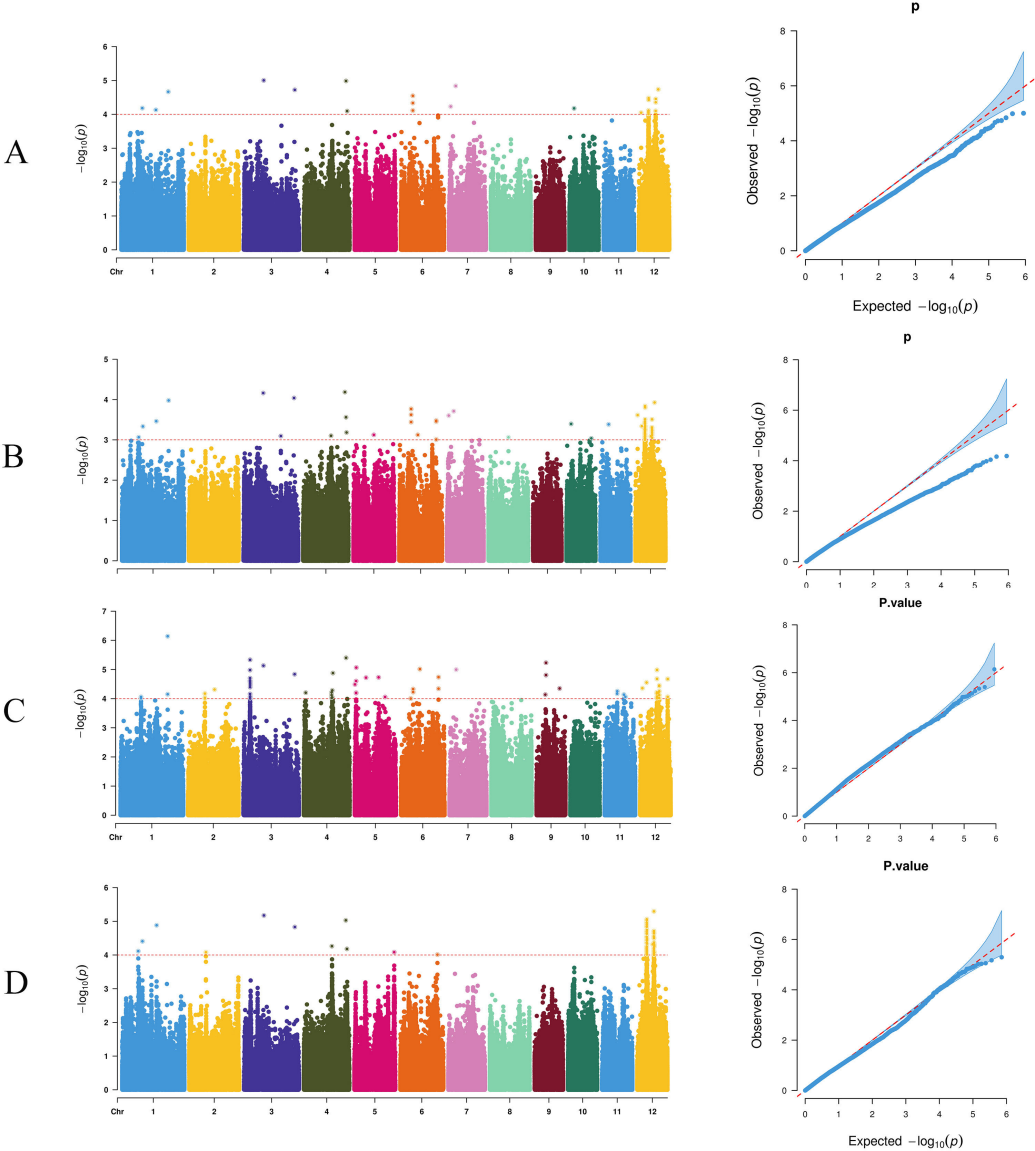
Manhattan plots and Q-Q plots for genome-wide association studies (GWAS) using four different methods. **(A)** TASSEL-GLM, **(B)** TASSEL-MLM, **(C)** EMMAX, and **(D)** GEMMA.

Using the GLM of TASSEL software, 27 SNP markers were detected genome-wide that were significantly associated with the target traits (threshold 1 × 10^-4^). The maximum number (6) of these significant loci mapped to chromosome 12, followed by 4 loci on chromosome 6 (**Figure 4A**). By contrast, using the MLM of TASSEL software, 89 significantly associated SNP markers (threshold: 1×10^-^³) were identified, with 63 loci predominantly located on chromosome 12 and 8 loci on chromosome 6 (**Figure 4B**). The EMMAX software's MLM yielded 79 significant SNP markers (threshold: 1×10^-4^), of which 63 loci were on chromosome 12 and 14 loci on chromosome 3 (**Figure 4C**). The GEMMA software's MLM identified 78 significant SNP markers (threshold: 1×10^-4^), with 67 loci primarily on chromosome 12 (**Figure 4D**). The detection results of all software showed significant enrichment on chromosome 12, suggesting that this chromosomal region may harbor major QTLs regulating the target trait.

**Figure 5:**
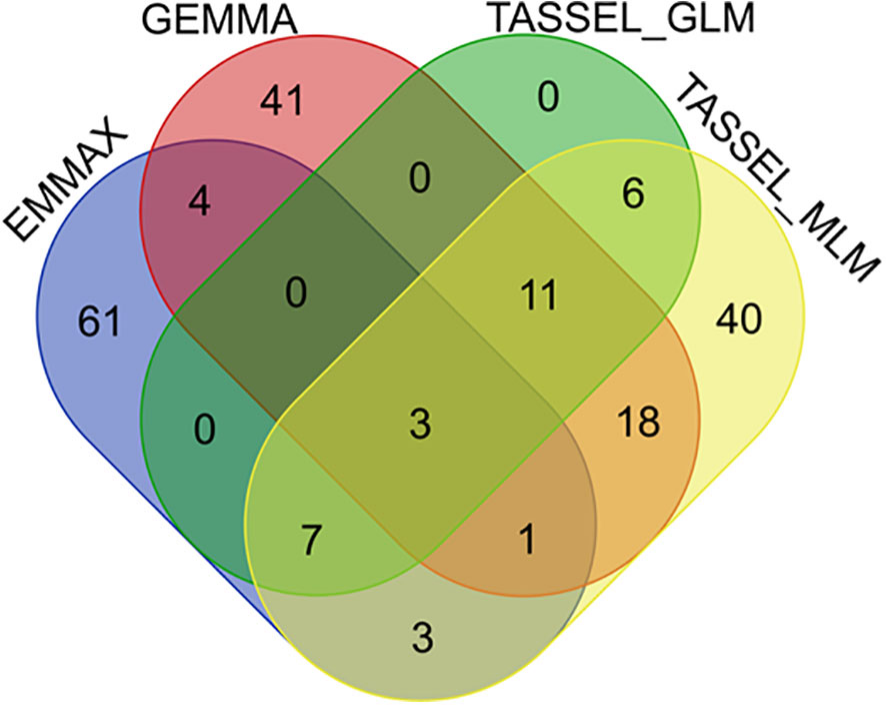
Venn diagram for screening significantly associated SNP markers.

**Figure 6:**
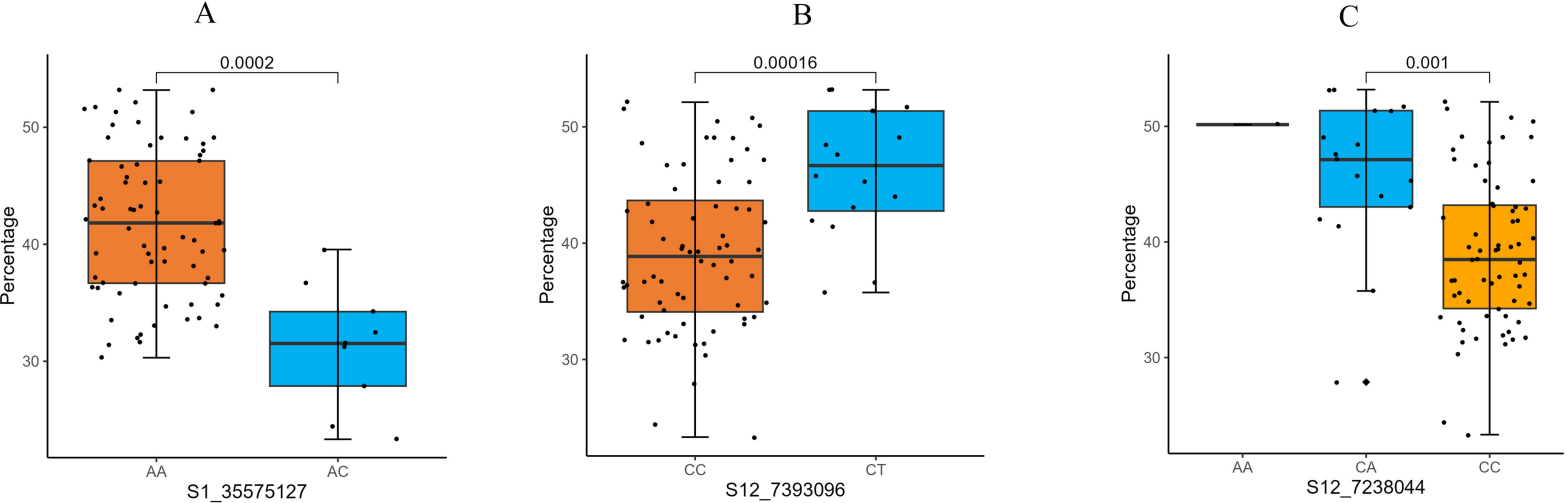
Distribution of sheath blight resistance phenotypes across genotypic groups at three GWAS-associated SNP loci. **(A)** Boxplot showing resistance percentage distribution for AA vs. AC genotypes at SNP locus S1_35575127. The AA genotype exhibits significantly higher resistance (p = 0.0002). **(B)** Resistance distribution for CC vs. CT genotypes at S12_7393096. The CT genotype shows significantly higher resistance (p = 0.0016). **(C)** Phenotypic variation between AA, CA, and CC genotypes at S12_7238044. The CA genotype is associated with the highest resistance level (p = 0.001). Each boxplot represents the median, interquartile range, and outliers; statistical significance was assessed using Welch’s t-test.

Subsequently, a Venn analysis was performed on the significant SNP markers resulting from the four models. As shown in **Figure 5**, three SNP markers were commonly identified by all four methods, located on chromosomes 3 (position: 38632300), 3 (position: 15089660), and 4 (position: 31894573), respectively. Twenty-two SNP markers were identified by three or more methods (**Figure 5**). For these 22 SNP loci, the genotypes of each sample and corresponding phenotypic information were extracted, and boxplots of genotype-phenotype relationships for each SNP locus were constructed. Taking three key SNP loci as examples, **Figure 6A** shows the distribution of lesion length ratio to leaf length for the S1_35575127 locus (Chr1: 35575127) on chromosome 1, with two genotypes (AA and AC). For the AA genotype, the median lesion length ratio was approximately 41.78%, indicating that most samples under this genotype exhibited lesion ratios concentrated around this level. The data distribution was relatively dispersed with a large box height, reflecting high variability in lesion ratios among AA genotype samples. The AC genotype had a median lesion length ratio of approximately 31.24%, significantly lower than that of the AA genotype, suggesting that *indica* rice carrying the AC genotype generally had lower lesion ratios and relatively stronger resistance to the ShB. The annotations above the two boxes in the figure indicate extremely significant differences in lesion length ratios between the two genotypes (P = 0.0002, P < 0.01); this demonstrates that the genotype of the SNP locus S1_35575127 is closely associated with ShB resistance in *indica* rice, with the AC genotype being favorable for enhancing resistance. The EF-hand domain-containing protein (EFh protein) regulated by this locus plays a critical role in crop disease resistance, primarily through the regulation of calcium signaling pathways (**Supplementary Table S6**). **Figure 6B** illustrates the distribution of lesion length ratio to leaf length for three genotypes (AA, CA, CC) at the S12_7238044 locus on chromosome 12. The AA genotype is represented by a single horizontal line, indicating a small sample size, with a lesion length ratio of approximately 50.15%. The CA genotype exhibited a median lesion length ratio of ~45.77%, with relatively concentrated data distribution and a small box height, suggesting low variability in lesion ratios among samples. The CC genotype had a median lesion length ratio of ~39.08%, which was lower than both the CA and AA genotypes, indicating that *indica* rice with the CC genotype generally exhibited lower lesion ratios and stronger ShB resistance. Although its data distribution was more dispersed (larger box height, higher variability), the annotations above the CA and CC genotype boxes (P = 0.0002, P < 0.01) confirmed extremely significant differences in lesion ratios between these genotypes. These results indicated that different genotypes at the S12_7238044 SNP locus are closely associated with ShB resistance in *indica* rice, with the CC genotype potentially being favorable for enhancing resistance. This locus is located in the regulatory region of the ATP-binding cassette (ABC) transporter G family member 43-like gene, which regulates ABC transporter families and participates in transmembrane transport of plant defense-related metabolites (**Supplementary Table S6**).

**Figure 6C** presents the distribution of lesion length ratios for two genotypes (CC, CT) at the S12_7393096 locus. The CC genotype had a median lesion length ratio of ~39.20%, with relatively dispersed data distribution and a large box height, reflecting high variability in lesion ratios among samples. The CT genotype exhibited a median lesion length ratio of ~46.23%, higher than that of the CC genotype, indicating that *indica* rice with the CT genotype had overall higher lesion ratios and weaker resistance. The annotations above the two boxes confirmed extremely significant differences in lesion ratios between CC and CT genotypes (P = 0.00016, P < 0.01). These findings demonstrated that the genotype at the S12_7393096 SNP locus is tightly linked to ShB resistance in *indica* rice, with the CC genotype potentially contributing to enhanced resistance. This locus is involved in regulating the expression of bi-functional inhibitor/plant lipid transfer protein/seed storage helical domain-containing protein (BiP/LTP/SSH), which contains lipid-binding motifs and antimicrobial peptide domains that may confer disease resistance by maintaining membrane integrity and direct action against pathogens.

### 3.5 Haplotype block analysis of consensus SNP-associated loci for ShB resistance

**Figure 7:**
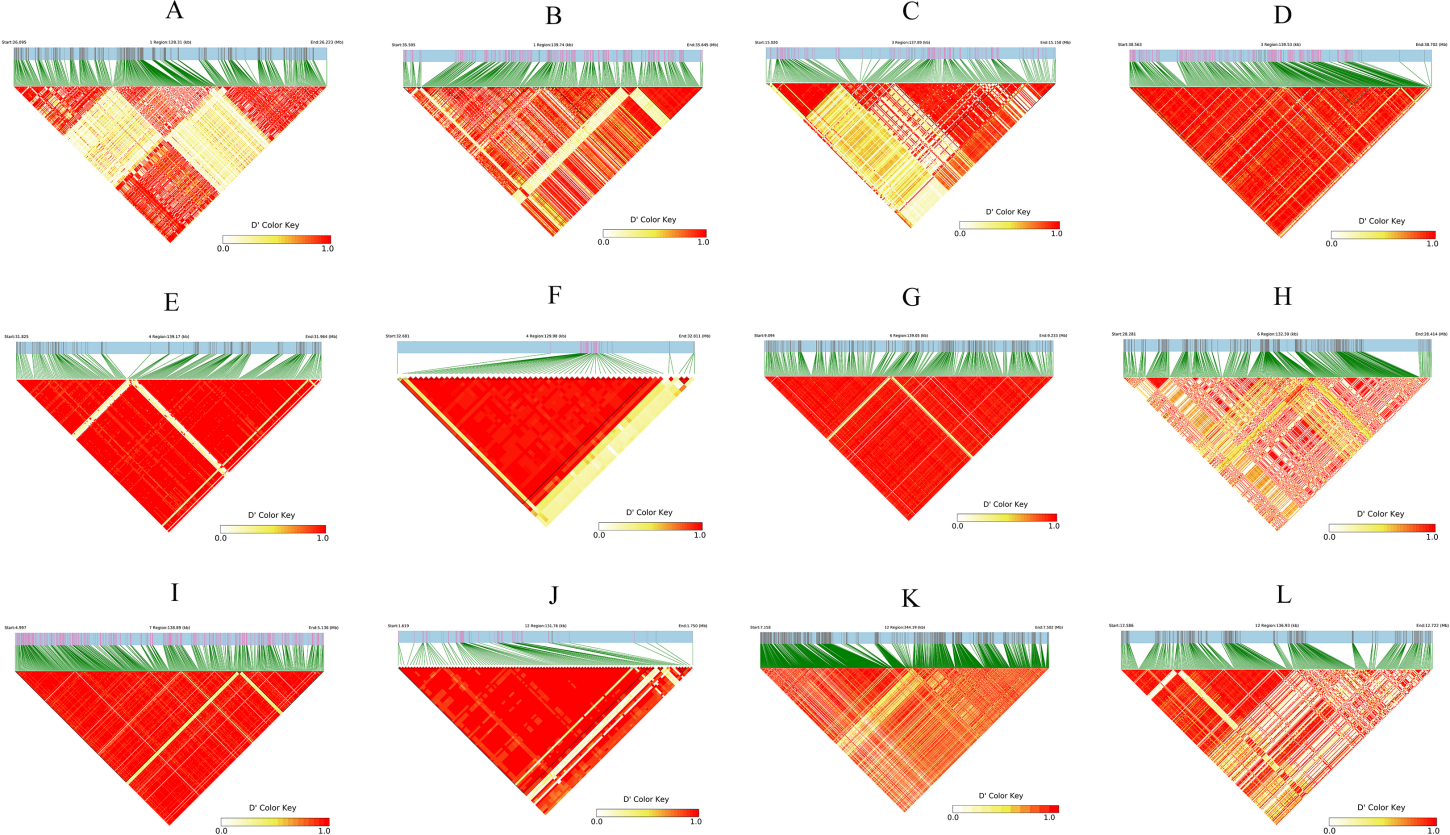
Linkage disequilibrium (LD) heatmaps of 70-kb genomic regions flanking 12 GWAS-associated SNPs. **(A–L)** Represent LD patterns for individual SNP loci identified in the GWAS analysis. Each heatmap displays the D' values of pairwise SNPs within a 70-kb window centered on the associated SNP (indicated at the top of each panel). The color gradient from light yellow to red reflects the degree of linkage disequilibrium (D'), ranging from 0.0 (no LD) to 1.0 (complete LD), with red indicating strong LD. Green lines denote the positions of SNPs, and red triangular regions highlight high LD blocks. Genomic coordinates (start and end positions) and region length are shown above each plot. These LD patterns help define haplotype blocks and identify candidate genes within the associated intervals.

**Figure 8:**
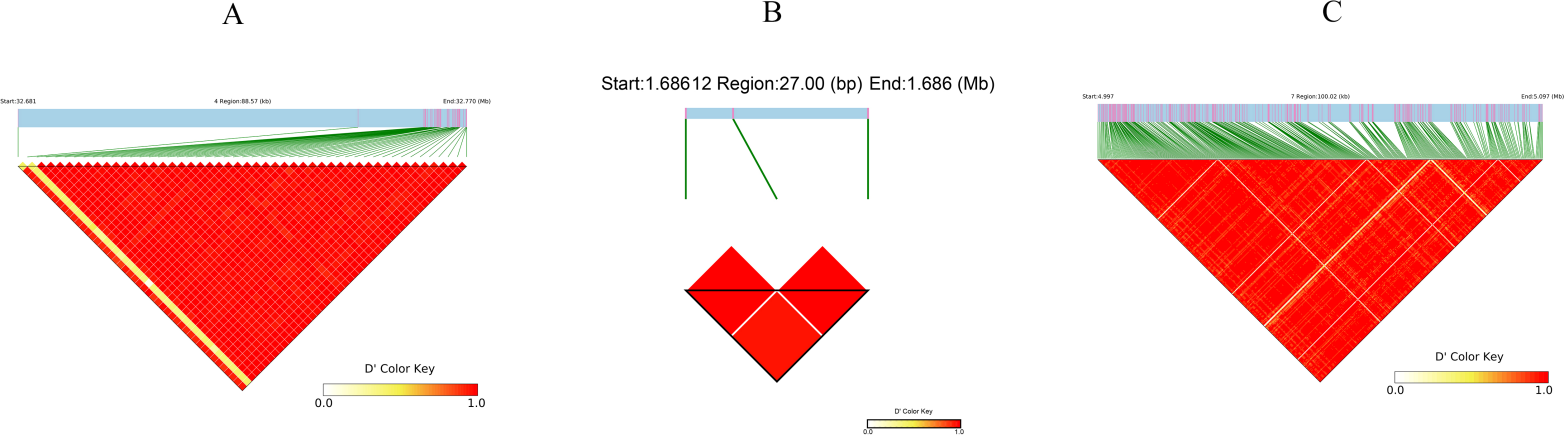
Linkage disequilibrium (LD) patterns in genomic regions harboring GWAS-associated loci. **(A)** LD heatmap of a 70-kb region surrounding a major resistance-associated SNP on chromosome 1 (S1_35575127), showing extensive high LD (D' > 0.8) across most SNP pairs, indicating strong haplotype block structure. **(B)** LD plot for a small 27-bp region near S12_7393096 on chromosome 12, where only two SNPs are present and exhibit complete LD (D' = 1.0). **(C)** LD heatmap of a 70-kb region flanking another significant SNP (S12_7238044), displaying moderate to high LD with several distinct blocks, suggesting potential functional haplotypes. In all panels, the color gradient from yellow to red represents D' values from 0.0 (no LD) to 1.0 (complete LD). Green lines indicate SNP positions, and diagonal bands reflect pairwise LD between SNPs.

Haplotype block analysis was performed by extending 70-kb regions flanking the 22 consensus SNPs. Firstly, we extracted the 70 kb intervals upstream and downstream of the 22 consensus SNPs. For regions containing multiple consensus SNPs, these intervals were merged. Using the LD-Block-Show software and based on VCF files, we generated linkage disequilibrium (LD) heatmaps, with the haplotype ranges simultaneously displayed within the heatmaps. We further filtered the regions in LD with the consensus SNP markers (i.e., the intervals containing consensus SNPs), which were defined as the three final critical blocks. LD-Block-Show generated heatmaps revealed 12 candidate intervals (**Figure 7**), including three on Chr 12, one on Chr 7, and two each on Chr 1, Chr 3, Chr 4, and Chr 6. Subsequent LD network analysis prioritized three critical blocks, which contained consensus SNPs (**Supplementary Table S7**): block4 (Chr 4:32,681,394–32,769,963), block7 (Chr 7:4,997,038–5,097,060), and block12 (Chr 12:1,685,784–1,685,811) (**Figure 8**). These regions exhibited strong LD persistence across multiple associated loci, suggesting potential regulatory hubs for ShB resistance. The critical LD block 4 (Chr 4:32,681,394–32,769,963) spans 88.6 kb, containing 49 SNPs. Functional annotation identified three candidate genes, including a leucine-rich repeat receptor-like kinase (LRR-RLK, At1g56130 ortholog) carrying pathogen recognition domains. This kinase, containing extracellular LRR motifs, is postulated to mediate pathogen-associated molecular pattern (PAMP) perception and subsequent immune signaling activation during *R. solani* infection, consistent with its role in basal resistance pathways.

The critical LD block 7 (Chr 7:4,997,038–5,097,060), spanning 100 kb, harbors 401 SNPs and 11 candidate genes, including nine with missense variants. Functional annotation highlights four mechanistically distinct candidates: an AAA+ ATPase domain protein implicated in cell wall remodeling through post-translational modification of acetylated proteins, potentially enhancing resistance against Gram-positive pathogens via lysozyme-mediated lysis (Jones et al., 2020); a glucose/sorbosone dehydrogenase hypothesized to modulate plant-pathogen interactions by altering cellular redox homeostasis, possibly through sorbosone-to-sorbosic acid conversion coupled with ROS signaling (Yang et al., 2021; Wang et al., 2023); a DDT domain-containing protein postulated to regulate immune signaling cascades via phosphorylation-dependent protein interactions (Wang et al., 2024); and a bHLH transcription factor demonstrating promoter-binding activity to defense-related genes, as evidenced by transcriptional activation assays (**Supplementary Table S8**) (Liao et al., 2025). These candidates collectively suggested that there are multi-layered defense mechanisms within this locus.

The critical LD block 12 (Chr 12:1,685,784–1,685,811) spans 28 bp and contains three SNPs, encompassing a single gene annotated as an uncharacterized protein. Despite limited functional evidence, this conserved genomic region is likely to harbor critical genetic determinants of ShB resistance, necessitating prioritized investigation through targeted mutagenesis and transcriptome profiling to elucidate its mechanistic role in disease defense pathways.

### 3.6 Expression patterns of candidate genes

**Figure 9:**
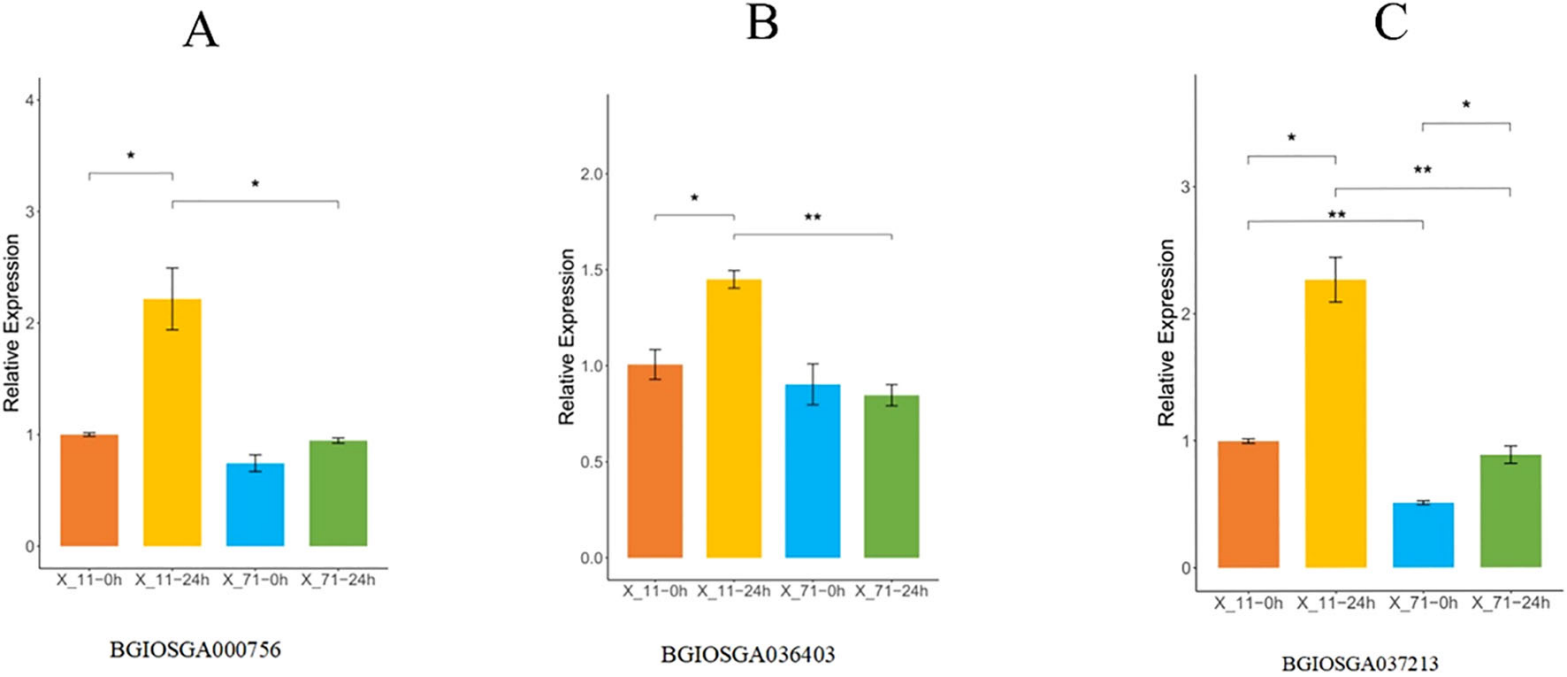
Time-course expression profiles of three candidate genes in resistant (X-11) and susceptible (X-71) rice lines following Rhizoctonia solani inoculation. **(A)** Relative expression of BGIOSGA000756 at 0 h and 24 h post-inoculation. Expression was significantly upregulated in X-11 at 24 h compared to the control (p < 0.05), while no significant change was observed in X-71. **(B)** Expression dynamics of BGIOSGA036403. The gene showed increased expression in X-11 at 24 h (p < 0.05), but remained stable in X-71. **(C)** Expression pattern of BGIOSGA037213, which exhibited strong induction in X-11 at 24 h (p < 0.01), whereas expression in X-71 remained low and unchanged. Data are presented as mean ± SD (n = 3). Statistical significance was assessed by Student’s t-test: **p < 0.01, *p < 0.05 versus the respective control (or X-71 at the same time point).

*BGIOSGA000756* encodes an EF-hand domain protein, a key component of the calcium signaling pathway, whose expression level directly influences the plant's early immune response to pathogens. qPCR results (**Figure 9A**) showed that at 0 hours post-inoculation (hpi), the relative expression level of *BGIOSGA000756* in the resistant line X-11 was 1.00 ± 0.02, higher than that in the susceptible line X-71 (0.74 ± 0.13), suggesting that regulatory sequence variations in this gene may confer a higher basal expression level in the resistant line even in the absence of pathogen stress. At 24 hpi, the expression level in X-11 was significantly upregulated to 2.19 ± 0.52, representing a more than two-fold increase compared to 0 hpi (p < 0.05), and was significantly higher than the level in X-71 at the same time point (0.94 ± 0.04, p < 0.05). In contrast, X-71 exhibited only a slight up-regulation (1.27-fold) after inoculation, which was not significant compared to its 0 hpi level (p > 0.05). These results indicate that variations in the regulatory region of *BGIOSGA000756* enhance its basal expression in the resistant line and facilitate further pathogen-induced activation, potentially strengthening calcium-mediated immune responses and contributing to the ShB resistance of X-11.

*BGIOSGA036403* encodes an ABC transporter involved in the transmembrane transport of defense-related metabolites; thus, its expression level potentially affects the accumulation efficiency of antimicrobial compounds. The qPCR analysis (**Figure 9B**) revealed that at 0 hpi, the relative expression in X-11 was 1.00 ± 0.13, higher than that in X-71 (0.90 ± 0.18), indicating a regulatory effect of non-coding sequence variations. At 24 hpi, the expression in X-11 increased sharply to 1.45 ± 0.08, a significant increase from its 0 hpi level (p < 0.05), and was significantly higher than the level in X-71 (0.84 ± 0.10, p< 0.01). Notably, the expression level in X-71 showed a decreasing trend after inoculation. These findings suggest that the resistant line X-11 can rapidly upregulate the expression of this ABC transporter upon pathogen infection, likely through cis-regulatory mechanisms, thereby accelerating the transport of antimicrobial metabolites to the infection site and/or enhancing the efflux and detoxification of fungal toxins, which aligns with the resistant phenotype of X-11.

*BGIOSGA037213* encodes a BiP/LTP/SSH protein implicated in maintaining membrane integrity and exerting direct antimicrobial activity; its expression is closely associated with cellular defense capacity under pathogen stress. According to qPCR data (**Figure 9C**), at 0 hpi, the relative expression level in X-11 was 1.00 ± 0.03, significantly higher than that in X-71 (0.51 ± 0.03, p<0.01), demonstrating that non-coding region mutations endow the resistant material with a stronger capacity for basal synthesis of this defense protein. At 24 hpi, the expression of *BGIOSGA037213* in X-11 was further upregulated to 2.27 ± 0.31 (more than two-fold increase compared to 0 hpi) and remained significantly higher than the level in X-71 (0.89 ± 0.12, p< 0.01). Although expression increased in X-71 after inoculation, it remained lower than the basal level observed in X-11 at 0 hpi. This result indicates that the resistant line X-11 activates the expression of *BGIOSGA037213* in response to pathogen challenge, potentially enhancing membrane stability and releasing antimicrobial peptides that directly inhibit the growth of *R. solani*. In contrast, the susceptible line X-71, likely due to weaker regulatory control of gene expression, fails to mount an effective membrane defense or antimicrobial response, leading to rapid disease progression.

Pearson correlation analysis was conducted between the resistance phenotypes of X_11 and X_71, and the expression levels of three genes (BGIOSGA000756, BGIOSGA036403, and BGIOSGA037213) detected at 24 hours post-inoculation with *Rhizoctonia solani* (the causal agent of sheath blight). The results showed that the correlation coefficients between the relative expression levels of BGIOSGA000756, BGIOSGA036403, and BGIOSGA037213 and lesion length were -0.97 (p = 0.0015), -0.93 (p = 0.0064), and -0.83 (p = 0.0398), respectively. These findings indicate a significant negative correlation between the aforementioned genes and lesion length—i.e., higher gene expression induced by the pathogen corresponds to shorter lesion length and stronger sheath blight resistance.

## 4 Discussion

The small sample size (n = 84) imposed inherent constraints on the statistical power of the GWAS, particularly for detecting low-frequency resistance alleles. To address this limitation, we implemented a multi-model framework that integrates GLM, MLM, EMMAX, and GEMMA analyses, complemented by Venn validation—a strategy that parallels approaches in maize smut resistance GWAS with constrained populations (Wang et al., 2012). Cross-model consensus analysis enhanced reliability through methodological triangulation, mirroring successful small-sample GWAS protocols in sweet potato that combined multi-locus models with stringent multiple testing corrections (Kim et al., 2023). These optimizations effectively balanced the risks of Type I and Type II errors while maintaining the potential for discovery, demonstrating the feasibility of robust association mapping in limited germplasm panels.

Small-sample GWAS requires stringent QC to prioritize high-confidence loci. Here, MAF filtering (>0.05) and LD-guided regional heritability estimation minimized spurious associations, paralleling approaches in large-scale *indica* rice GWAS (Zhang et al., 2019). While cohort size constraints remain inherent, as demonstrated by Fu et al (Fu et al., 2022). in their multi-year validation in 150 rice accessions, integrating diverse genetic backgrounds (π = 0.0028) and kinship-adjusted models enhanced detection accuracy, reflecting Chen et al.'s (Chen et al., 2019) subpopulation optimization strategy. Future efforts should expand germplasm diversity and incorporate multi-environment trials to dissect G × E interactions, thereby advancing translational breeding for sheath blight resistance. ABCG transporters orchestrated multi-layered defense mechanisms against *R. solani* infection. Primarily, they mediate efflux detoxification of fungal toxins (e.g., CWDEs) through substrate-specific transport, limiting cellular damage (Costanzo et al., 2011; Jia and Gealy, 2018). Alongside, these proteins regulate systemic immunity by spatially coordinating the translocation of defense hormones (SA/JA), thereby priming the expression of pathogenesis-related genes (Qin et al., 2023). Beyond their classical roles, ABCGs facilitate the apoplastic accumulation of phenylpropanoid-derived antimicrobials, including phenolics and lignin precursors, through transmembrane transport, thereby establishing dual chemical-physical barriers against pathogen invasion (Elsharkawy et al., 2022; Hunjan et al., 2022). Emerging evidence suggests non-canonical functions in pathogen-associated molecular patterns (PAMP) perception, where ABCG-mediated recognition of fungal extracellular polysaccharides (EPS) may initiate innate immune signaling, unveiling novel dimensions in plant-pathogen interface studies (PPIS) (Ma et al., 2020).

Calmodulin-like proteins (CMLs), a pivotal EF-hand domain protein family in plants, orchestrate the stress responses through calcium signaling (Zeng et al., 2023). Rice *OsCML3* (*Os01g0765600*) exemplifies this mechanism, exhibiting pathogen-induced upregulation and interacting with high-mobility group proteins (HMGB) through C-terminal extension to activate defense gene networks (Zeng et al., 2023). Calcium-dependent conformational changes in *OsCML3* modulate downstream signaling, including MAPK cascades and redox homeostasis. Specifically, *OsCML3* balances NADPH oxidase-mediated ROS production with antioxidant activity, mitigating oxidative stress during infection (Liu et al., 2022; Zeng et al., 2023). This dual regulatory capacity positions CMLs as hubs integrating calcium and redox signaling. Notably, a coding SNP (A2WVE9) in *OsCML3* alters structural dynamics and sub-cellular localization, potentially fine-tuning immune responses (Zeng et al., 2023; Yang et al., 2024), and offers targets for breeding calcium-regulated disease resistance.

Plant lipid transfer proteins (LTPs) are small, alkaline proteins widely present in higher plants, with multifunctional roles that include phospholipid transfer, cutin formation, signal transduction, and defense mechanisms against disease resistance (Collinson et al., 2024; Shang et al., 2025). Studies have shown that LTPs play essential roles in plant resistance to pathogen infection. For example, LTPs in rice enhance resistance to bacterial diseases by regulating cell membrane stability and signal transduction (Schierenbeck et al., 2021). Additionally, LTPs can interact with ROS-scavenging enzymes such as ascorbate peroxidase (APX) to regulate ROS levels and enhance plant defense against pathogens (Schierenbeck et al., 2021). In rice, overexpression of LTP significantly enhances resistance to bacterial blight, while LTP deficiency compromises this resistance, underscoring the critical role of LTPs in rice disease resistance (Schierenbeck et al., 2021). LTPs can interact with pathogenesis-related (PR) proteins, which were produced by plants in response to pathogen attack, exhibiting antibacterial and antiviral functions; their synergistic actions more effectively inhibit pathogen invasion and reproduction (Kumar et al., 2025). Bifunctional inhibitors (BiPs) act as molecular chaperones, primarily participating in protein folding and endoplasmic reticulum stress responses. In plant disease resistance, BiPs enhance pathogen defense by regulating protein homeostasis and signal transduction. Past studies have shown that BiP expression in rice is closely associated with disease resistance (Park et al., 2010; Park et al., 2025). For example, BiP improves rice resistance to fungal diseases by regulating endoplasmic reticulum stress responses, with its expression level significantly upregulated after pathogen infection, indicating its essential role in disease resistance responses (Das et al., 2024). Furthermore, BiPs form complex defense networks through interactions with other disease resistance-related proteins, further enhancing plant disease resistance (Jain and Khurana, 2018; Zribi et al., 2024). Seed storage helical domain-containing proteins (SSHs), as proteins with specific domains, mainly participate in the accumulation and regulation of seed storage substances (Antonets et al., 2020). In disease resistance research, SSHs indirectly influence plant disease resistance by regulating storage substances in seeds (Shewry et al., 1995; Schwarzenbach, 2013). For example, the SSH expression in rice is closely correlated with the content of storage substances in seeds, which is essential for plant disease resistance (Li et al., 2018). It is reported that SSH expression levels in rice are significantly upregulated after pathogen infection, indicating their essential role in disease resistance responses (Li et al., 2018). Additionally, SSHs form complex defense networks through interactions with other disease resistance-related proteins, further enhancing plant disease resistance (Xiong et al., 2023). Future research could further explore the interactions among these proteins and their specific mechanisms in disease resistance, for example, by utilizing gene editing technologies to investigate their functions in rice disease resistance and their interactions with other proteins involved in disease resistance. Moreover, multi-omics analyses could reveal the global regulatory networks of these proteins in disease resistance.

The LRR-RLKs are transmembrane proteins comprising an extracellular LRR domain, a transmembrane domain, and an intracellular kinase domain. The LRR domain is responsible for recognizing specific ligands (such as pathogen-derived molecules), while the kinase domain activates downstream signaling pathways through phosphorylation (Xu et al., 2023; Xu et al., 2024). In rice, the LRR-RLK family comprises numerous members involved in various biological processes, including development, hormone signaling, and disease resistance responses (Block et al., 2021; Durgadevi et al., 2021). The rice LRR receptor-like protein (*OsRLP1*) and its adaptor kinase *OsSOBIR1* jointly mediate resistance to viral infections (Zhang et al., 2021). Through high-throughput sequencing and transgenic rice experiments, researchers found that *OsRLP1* and *OsSOBIR1* are significantly upregulated after viral infection and can activate plant immune responses, revealing the vital role of LRR-RLKs in plant antiviral defense (Zhang et al., 2021).

In this study, we analyzed the expression patterns of three candidate genes (*BGIOSGA000756*, *BGIOSGA036403*, and *BGIOSGA037213*) associated with GWAS loci located in regulatory or intronic regions. Under non-infected conditions, the resistant line X-11 exhibited higher basal expression levels of all three genes compared to the susceptible line X-71. This suggests that non-coding variations at these GWAS loci may enhance promoter activity or transcription efficiency, thereby providing a pre-activated defense foundation in the resistant material even in the absence of pathogen stress. At 24 hours post-inoculation (hpi) with *R. solani*, all three genes were significantly induced (1.4- to 2.2-fold) in X-11, whereas their induction was markedly weaker or even decreased in X-71. This result further indicates that these GWAS loci are involved not only in regulating basal expression but also in activating the pathogen-induced expression pathway. We speculate that the regulatory variations may contain pathogen-responsive elements that are specifically functional in the resistant genotype, enabling efficient activation of defense responses upon pathogen recognition. Collectively, these non-coding variations likely contribute to the resistant phenotype by modulating gene expression levels rather than altering protein sequences.

The high expression of these genes in X-11 likely underpins its resistance mechanism through distinct roles. *BGIOSGA000756*, encoding an EF-hand domain protein, may facilitate the rapid transmission of pathogen invasion signals through calcium signaling, potentially activating downstream MAPK cascades and ROS bursts, thereby initiating subsequent defense reactions. The elevated expression of *BGIOSGA036403*, an ABC transporter, could promote the transport of antimicrobial metabolites to the infection site, forming a chemical barrier, while also effluxing fungal toxins to reduce cellular damage. Meanwhile, *BGIOSGA037213*, a BiP/LTP/SSH protein, may enhance resistance by maintaining membrane integrity against pathogen degradation and potentially directly inhibiting *R. solani* hyphal growth through the production of antimicrobial peptides. While this study advances our understanding of ShB resistance genetics, several limitations warrant consideration. The modest cohort size may limit detection of low-frequency resistance alleles, and reliance on controlled *in vitro* assays may not fully capture field resistance dynamics. Future investigations should incorporate multi-environment trials to validate ecological relevance. Although candidate genes were prioritized, functional validation through CRISPR-Cas9 editing and mechanistic studies (e.g., transcriptomics, yeast two-hybrid assays) remains imperative to delineate their roles in defense signaling. Integrated multi-omics approaches (proteomics/metabolomics) could further unravel regulated pathways and protein interaction networks, advancing resistance breeding strategies.

Although this study enhances our understanding of the genetic basis of sheath blight (ShB) resistance, several limitations should be noted. Future work should prioritize the functional characterization of the three core candidate genes (*BGIOSGA000756*, *BGIOSGA036403*, *BGIOSGA037213*) and 22 consensus SNPs identified here, to elucidate their roles and potential utility in improving ShB resistance in indica rice. To this end, knockout lines for each candidate gene could be generated in the resistant accession X-11, while overexpression lines could be created in the susceptible accession X-71. Using the *in vitro* leaf segment inoculation method developed in this study, along with whole-plant inoculation assays under greenhouse conditions, comparative phenotypic analyses can be conducted to assess the impact of gene knockout or overexpression on resistance-related traits, such as lesion length ratio and lesion height. In parallel, yeast two-hybrid assays could be employed to identify interacting partners of the candidate proteins, helping to decipher their molecular mechanisms in calcium signaling, antimicrobial metabolite transport, or maintenance of cell membrane integrity.

Furthermore, the 22 consensus SNPs could be converted into molecular markers for high-throughput genotyping across a broader collection of indica rice germplasm. By combining such marker screening with multi-environment field trials, researchers could validate the associations between these SNPs and ShB resistance. This would ultimately provide valuable genetic resources and practical tools for marker-assisted breeding of ShB-resistant indica varieties. Together, these approaches would address the current limitations in establishing gene causality and translating laboratory findings into field applications, thereby accelerating the development of ShB-resistant *indica* rice through improved breeding efficiency.

## 5 Conclusions

This study employed GWAS on 84 *indica* rice accessions to dissect the genetic architecture of ShB resistance. Multi-model integration (GLM/MLM/EMMAX/GEMMA) and Venn consensus analysis identified 22 robustly associated SNPs, with significant enrichment on chromosome 12, suggesting major-effecting QTLs. Haplotype-based fine mapping delineated three critical LD blocks harboring 15 candidate genes, including ABC transporters, calmodulin-like proteins, lipid transfer proteins, and LRR receptor-like kinases. Functional annotation associates these candidates with defense metabolite trafficking, calcium signaling modulation, and pathogen recognition, key mechanisms underpinning basal immunity. The findings demonstrate that non-coding variations in three GWAS-linked candidate genes enhance disease resistance in rice line X-11 by conferring strong basal and pathogen-induced expression, as validated by qPCR analysis following *R. solani* challenge. The identified loci establish a genetic framework for molecular breeding strategies. Future efforts should expand germplasm diversity, incorporate multi-environment trials, and deploy CRISPR-Cas9-mediated functional validation to accelerate the development of ShB-resistant *indica* varieties.

## Data Availability

Data generated or analyzed during this study are included in this published article and its supplementary information files. The datasets presented in this study are available in online repositories. The names of the repository and accession number(s) can be found below: NCBI https://www.ncbi.nlm.nih.gov/, Accession number: PRJNA1358769.
