# Supplementary figures and images for: Mining SNP loci and candidate genes for sheath blight resistance in Indica rice using genome-wide association studies
Source: Front Plant Sci. 2025 Dec 18;16:1718389. doi: 10.3389/fpls.2025.1718389 (PMC12756348; doi:10.3389/fpls.2025.1718389)

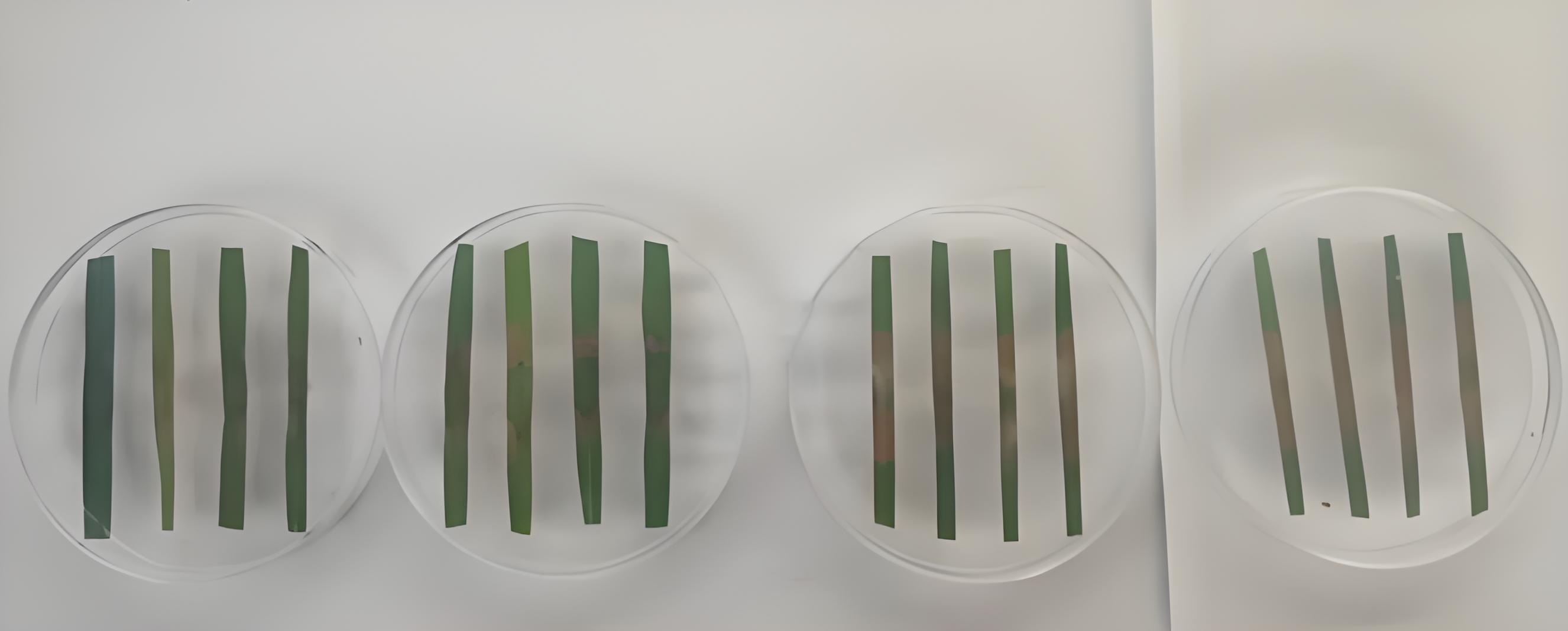

Supplement: Supplementary file 1 [file Image1.jpeg]

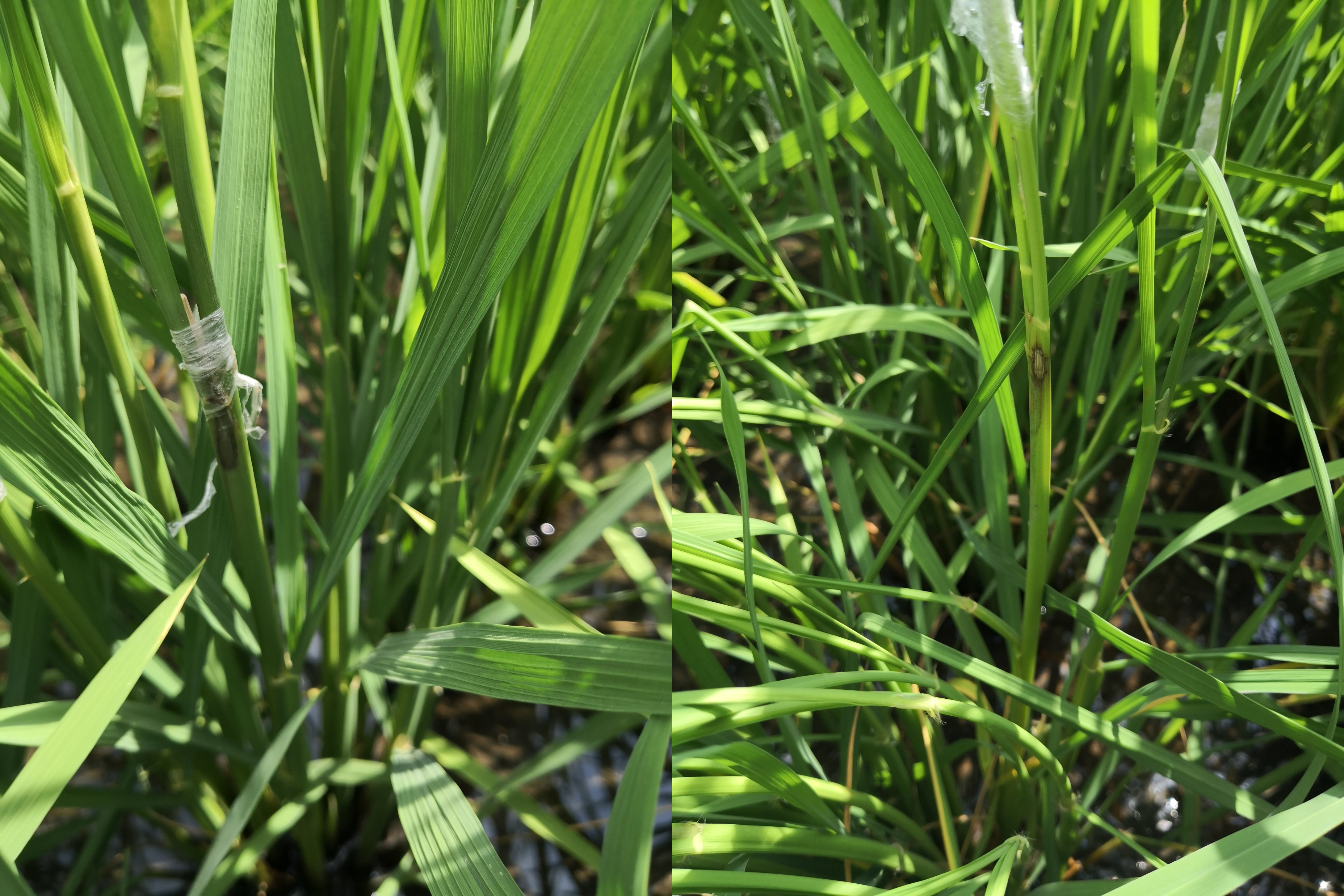

Supplement: Supplementary file 2 [file Image2.png]
